# Heterologous Synthesis of Monacolin J by Reconstructing Its Biosynthetic Gene Cluster in *Aspergillus niger*

**DOI:** 10.3390/jof8040407

**Published:** 2022-04-16

**Authors:** Xu Zeng, Junwei Zheng, Feifei Lu, Li Pan, Bin Wang

**Affiliations:** 1 School of Biology and Biological Engineering, South China University of Technology, Guangzhou Higher Education Mega Center, Panyu Town, Guangzhou 510006, China; 201920146393@mail.scut.edu.cn (X.Z.); 201710106321@mail.scut.edu.cn (J.Z.); 202120149694@mail.scut.edu.cn (F.L.); 2 Guangdong Provincial Key Laboratory of Fermentation and Enzyme Engineering, Guangzhou 510006, China

**Keywords:** Monacolin J, heterologous synthesis, reconstruction of the biosynthetic gene cluster, CRISPR/Cas9 homology-directed recombination (CRISPR-HDR), *Aspergillus niger*

## Abstract

Monacolin J (MJ), a key precursor of Lovastatin, could synthesize important statin drug simvastatin by hydrolyzing lovastatin and adding different side chains. In this study, to reduce the cumbersome hydrolysis of lovastatin to produce MJ in the native strain *Aspergillus terreus*, the MJ biosynthetic pathway genes (*lovB*, *lovC*, *lovG,* and *lovA*) were heterologously integrated into the genome of *Aspergillus. niger* CBS513.88 with strong promoters and suitable integration sites, via yeast 2μ homologous recombination to construct expression cassettes of long-length genes and CRISPR/Cas9 homology-directed recombination (CRISPR-HDR) to integrate MJ genes in the genome of *A. niger*. RT-PCR results proved that pathway synthesis-related genes could be heterologously expressed in *A. niger*. Finally, we constructed an engineered strain that could produce monacolin J, detected by LC-HR-ESIMS (MJ, 339.22 [M-H]^+^). The yield of MJ reached 92.90 mg/L after 7-day cultivation. By optimizing the cultivation conditions and adding precursor, the final titer of MJ was 142.61 mg/L on the fourth day of fed-batch cultivation, which was increased by 53.5% compared to the original growth conditions. Due to the wide application of *A. niger* in industrial fermentation for food and medicine, the following work will be dedicated to optimizing the metabolic network to improve the MJ production in the engineered strain.

## 1. Introduction

Monacolin J (MJ) is a key precursor of statin drugs like lovastatin and simvastatin that can competitively inhibit the activity of hydroxymethyl glutarate coenzyme A (HMG-CoA) reductase [1], thereby blocking cholesterol biosynthesis. MJ is of great importance in the production of cholesterol-lowering drugs. At present, the industrial production of MJ is mainly accomplished by alkali hydrolysis of lovastatin, causing considerable financial costs and environmental burdens. Nowadays, the biosynthesis of MJ is regarded as a sustainable strategy, compared with chemical methods. Lv et al. directly in vivo hydrolyzed the synthesized lovastatin into MJ by heterologously expressing lovastatin hydrolase PcEST in *Aspergillus terreus*, and the final hydrolysis rate of lovastatin reached 95% [2]. However, the residual lovastatin still presented difficulties for the purification of MJ. The direct biosynthesis of MJ could provide a solution to solve the above problem [3].

**Figure 1:**
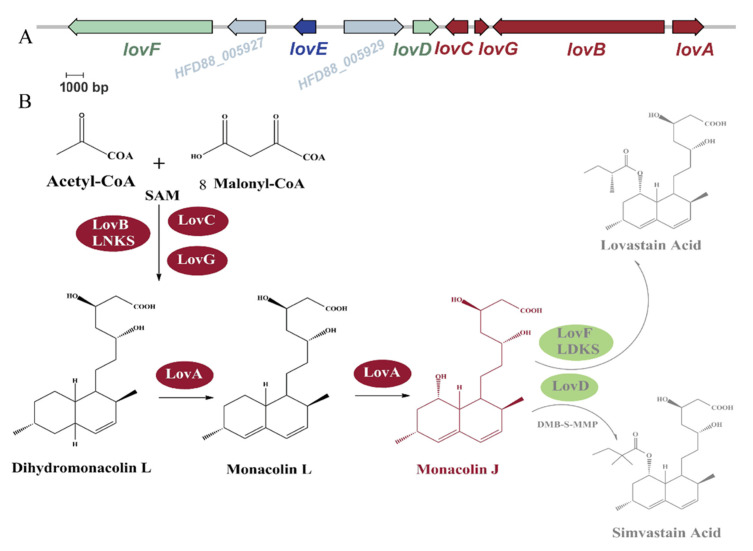
Scheme of the lovastatin gene cluster and biosynthetic pathway. (**A**) Lovastatin gene cluster in *Aspergillus terreus*. Genes marked in red are required for the synthesis of MJ. (**B**) Lovastatin acid and simvastatin acid biosynthetic pathway. LovB (lovastatin nonaketide synthase, LNKS) and LovC, combined with LovG, are responsible for the synthesis of dihydromonacolin L (DML); then, DML was modified by LovA through continuous two-step oxidization to produce monacolin L (ML) and monacolin J (MJ). The α-methylbutyryl side chain is synthesized by LovF (lovastatin diketosynthase, LDKS) and is transferred to MJ by LovD to produce lovastatin (LV) acid. LovD could also catalyze MJ with an acyl donor DMB-SMMP to produce simvastatin. SAM: S-adenosyl-L-methionine, DMB-S-MMP: α-dimethylbutyryl-S-methyl-mercaptopropionate.

The native biosynthetic pathway of MJ in *A. terreus* is a part of the synthetic pathway of lovastatin, and the required enzymes are encoded in the *lov* gene cluster [4] (Figure 1A). The genes *lovC*, *lovG*, *lovB*, and *lovA* in the *lov* cluster are for the synthesis of MJ, while *lovF* and *lovD* catalyze the subsequent steps to produce lovastatin [5], and *lovD* was also able to catalyze the direct acylation of MJ by the novel thioester α-dimethylbu-tyryl-S-N-acetylcysteamine (DMB-S-NAC) as the acyl donor to produce simvastatin [6] (Figure 1B). LovE, encoded by *lovE*, is a GAL4-like transcriptional factor protein that could control the expression level of *lov* genes [7]. Under the joint catalytic activities of LovBCG, dihydromonacolin L(DML) is synthesized as the first product, using acetyl-CoA, 8 malonyl-CoA, and SAM as building blocks, where LovB (lovastatin nonaketide synthase, LNKS), a polyketide synthase I, catalyzes the iterative initial key stage of lovastatin synthesis, LovC as a separate enoylreductase constructs the 2-methy butyryl side chain [8], and LovG as a thioesterase releases DML [9]. Subsequently, LovA, a cytochrome P450 oxidase, catalyzes two-step oxidation to produce monacolin L and MJ from DML [10]. The production of natural products in fungi is generally time- and labor-consuming, meanwhile the initial titers in native producing strains are often considered low. Heterologous expression of target synthetic pathway in chassis microorganisms is a promising strategy for the production of bioactive compounds. Liu et al. used the methanol-inducible promoter *Aox1* to reconstitute the lovastatin pathway in *Pichia pastoris*, and demonstrated the heterologous biosynthesis of natural products from the one-carbon substrate methanol and detected the titer of MJ to 60 mg/L [11]. Tang et al. reconstructed the lovastatin biosynthetic pathway of *A. terreus* in *Saccharomyces cerevisiae* by introducing six heterologous biosynthetic genes into *S*. *cerevisiae* and adding acyl donor DMB-SMMP, finally synthesizing simvastatin, with a yield reaching 55 mg/L [12]. However, the genetic distance between native and host strains should be taken into account [13]. Compared to yeast, *A. niger* and *A. niger* and *A. terreus* are both filamentous organisms belonging to the *Aspergillus* genus *Aspergillus* genus, and *A. niger* has been widely used as an industrial model microorganism for the production of enzymes like glucoamylase and organic acids such as citric, gluconic and oxalic acids [14]. The *Aspergillus* genus is also of great interest in the production of secondary metabolites, for it possesses various efficient genetic toolkits and a clear genetic background. Filamentous fungi genomes, containing diverse secondary metabolite biosynthetic gene clusters (BGCs) and an enormous reservoir of untapped chemical potential [15], are also of great interest in the production of secondary metabolites. In the case of MJ, which is natively produced in *A. terreus*, *A. niger* was a reasonable host for heterologous production although the construction of long-fragment genes, and limitation in selection markers are still challenges that should be considered.

In this study, based on yeast 2μ homologous recombination technology [16] and CRISPR-HDR technology for site integration [17], the obstacles of expressing long-fragment genes and reuse of the selection marker *pyrG* are addressed in detail. We successfully constructed a de novo MJ synthetic pathway in *A. niger*. Then, by optimizing the cultivation parameters (pH and carbon source), the titer of MJ was improved in fed-batch cultivation. The reconstituted MJ biosynthetic pathway in *A. niger* could be used to continue the exploration of statin derivatives using MJ as the precursor.

## 2. Materials and Methods

### 2.1. Strains and Culture Conditions

*Escherichia coli* Mach1 T1 was used for plasmid cloning, and *E. coli* transformants were cultivated in the Luria-Bertani (LB) medium (1% tryptone, 0.5% yeast extract, and 1% NaCl) with 100 μg/mL ampicillin at 37 °C. *Saccharomyces cerevisiae* BY4741 (MATa his3Δ1, leu2Δ0, met15Δ0, ura3Δ0) [18] was cultivated in YPD medium (yeast-peptone-dextrose extract, containing 2% glucose, 1% peptone, 1% yeast extract) at 30 °C. *S. cerevisiae* transformants were cultivated in SD-Ura medium (Synthetic Dropout Medium–Uracil), containing 2% glucose, 0.67% YNB (Yeast Nitrogen Base Without Amino acids), 0.12% DO Supplement–Uracil. *Aspergillus niger* CBS513.88 (Δ*kusA*, Δ*pyrG*) and all constructed strains used in this study are shown in Table 1. Strains were cultivated on potato dextrose agar (PDA) medium for 7 days at 30 °C to collect spores. Culture medium for *A. niger* transformation was the dextrose-peptone-yeast extract (DPY) medium, containing 2% glucose, 1% peptone, 0.5% yeast extract, 0.1% K_2_HPO_4_ and 0.05% MgSO_4_·7H_2_O.

*A. niger* medium for MJ production contains 4% glucose, 2% peptone, 0.2% NaNO_3_, 0.2% K_2_HPO_4_, and 0.1% MgSO_4_·7H_2_O, pH 6.5. For cultivation medium with different carbon source (such as lactose, xylose, glycerol, starch, and maltose), glucose was replaced with the same amount of the other carbon source, and the rest was the same. The fed batch of supplement maltose was conducted by adding 5 mL 0.4 g/mL sterilized maltose solution to each conical flask on the 4th day of cultivation.

### 2.2. Recombinant Plasmid Construction

The ORF (open reading frame) of *lov* genes required for biosynthesis of MJ was amplified using the genomic DNA of *Aspergillus terreus* ATCC 20542 as template and NEBuilder Hifi DNA Assembly Master Mix (Catalog #E2621L) according to the manufacturer’s protocol. The expression vectors of *lovC, lovG,* and *lovA* were constructed using *E. coli* Mach1 T1 as the cloning host, while the expression vector of *lovB* was constructed in *S. cerevisiae* using 2μ homologous recombination technology (described in Section 2.3).

For the expression vector of *lovC/G/A*, the genetic elements were first assembled into two or three separate DNA fragments by overlapping PCR. Then, DNA fragments were cloned into pMD18(+) T-vector via in-fusion cloning, with the molar ratio of fragments to vector being 5:1. The in-fusion mixture was transformed into *E. coli* Mach1 T1 and positive transformants were selected. Plasmids were extracted using a Plasmid kit (Magen, Guangzhou, China) and confirmed by sequencing. The final recombinant plasmid should contain three functional parts: the upstream fragment of the knock-in site, the expression cassette of *lov* gene, and the downstream fragment of the knock-in site. The construction strategy of each plasmid was described in Supplemental Table S1. Primers used in this study was described in Supplemental Table S2.

### 2.3. Assembly of Long-Length PKS Gene via Yeast 2μ Homologous Recombination

As for long-length PKS gene *lovB* was PCR amplificated by two parts with 50 bp overlapping, then the donor DNA fragments (promoter P*glaA*, terminator T*tef*, selection mark *pyrG_An_*, integrated homology arm) and yeast expression vector pYEP352 (digested with *XbaI*), were co-transformed in yeast by PEG/LiAc method [19]. Linear plasmid and donor DNA fragments were added in a molar ratio of 1:3:3 to carry out yeast transformation. Following transformation, yeast spheroplasts were regenerated and selected on complete supplemental medium without Uracil (SD-ΔUra) agar plates for 3 days at 30 °C.

### 2.4. Aspergillus Niger Genetic Manipulation via CRISPR/Cas9 Technique

The sgRNA of specific site knock-out vectors for site integration of *A. niger* was designed on the basis of predictions by the website: http://www.rgenome.net/cas-offinder/ (accessed on 18 July 2021). The expression vectors of *Streptococcus pyogenes* Cas9 protein used in this experiment for integrating the *pyrG_An_* (Orotidine-5′-phosphate decarboxylase An12g03570), *aamA* (acid alpha-amylase An11g03340), and *amyA* (neutral amylase An05g02100) genes were reconstructed based on the pFC332 plasmid including Cas9 and basic template of sgRNA-cloning cassette [20]. The plasmids used for site integration are listed in Supplemental Table S3.

For the genetic transformation of *A. niger*, the protoplast transformation method was used as described previously [21]. *A. niger* spores were inoculated in liquid DPY medium and cultured in shake flasks for 2 days, after enzymatic hydrolysis of mycelium [22], the purified protoplasts were collected, and the donor fragment and knock-out plasmid were added to the protoplasts, followed by the corresponding selection marker additives, spread on the selection plate, incubated at 30 °C for 5–10 days. LovB expression cassette P*glaA*-*lovB*-T*tef*-*pyrG_An_* was transformed into *A. niger* CBS513.88 (Δ*kusA* Δ*pyrG*) with 1500 bp upstream and downstream homologous fragment to replace the glucoamylase site (An03g06550). The obtained transformant was named as CBS-LovB. The *pyrG_An_* gene encodes *A. niger* 5′-phosphodecarboxylase, and strains lacking this enzyme cannot grow on media without uracil. *A. niger* strain CBS-LovB restored *pyrG* prototrophy. In the second round of transformation, via CRISPR-HDR technology, the plasmid PFC332-*pyrGAn*-*hygB* (contains sgRNA for *pyrG* gene and Cas9 expression cassette) and the linearized plasmid pM18T-Up*_pyrG_*-P*_gpdA_*-*lovC*-T*tef*-Dn*_pyrG_* were co-transformed to integrate *lovC* expression cassette into the *pyrG_An_* site in the genome of *A. niger* CBS-LovB, the positive transformant CBS-LovBC was selected by hygromycin B resistance and *pyrG* auxotrophy. In the third round of transformation, in the same way, the linearized plasmid pM18T-Up*_aamA_*-P*_tef_*-*lovG*-T*_tef_*-*pyrG_An_*-Dn*_aamA_* was co-transformed with gRNA vector PFC332-*aamA*-*hygB* (contains sgRNA for amylase gene *aamA* and Cas9 expression cassette) to integrate *lovG* expression cassette into the site of amylase *aamA* and restore *pyrG* prototrophy; the obtained transformant was named CBS-LovBCG. Finally, the linearized plasmid pM18T-Up*_amyA_*-P*_tef_*-*lovA*-T*_tef_*-Dn*_amyA_* was co-transformed with gRNA vector PFC332-*amyA*-*hygB* (contains sgRNA for amylase gene *amyA* and Cas9 expression cassette) to integrate *lovA* expression cassette in the neutral amylase sites (*amyA*, An05g02100). *A. niger* transformant CBS-LovBCGA was selected on the basis of hygromycin B resistance (Figure S1).

The selection plate CD medium (2.0% glucose, 0.3% NaNO_3_, 0.2% KCl, 0.05% MgSO_4_·7H_2_O, 0.001% FeSO_4_·7H_2_O, 0.1% KH_2_PO_4_, pH 5.5). The solid medium was supplemented with 2% agar, the soft agar was supplemented with 0.5%, and 10 mM Uracil was added to maintain nutrition or 100 μg/mL hygromycin B was added to maintain selection.

The *A. niger* transformants were identified by PCR amplification and the PCR products were subjected to Sanger sequencing. All primers used in this study are listed in Supplemental Table S2.

### 2.5. HPLC Detection and LC-MS/MS Analysis of MJ

To examine MJ production, PDA medium was cultured 7 days to collected spores, 1 × 10^7^ spores of *A. niger* transformants were inoculated into 100 mL medium in 250 mL Erlenmeyer flask with a few glass beads. The culture was incubated at 30 °C at 220 rpm on a rotary shaker for 7 days. For detection, the supernatant of the cultivation broth was taken, as well as an equal volume of 0.2 M NaOH; 10 volumes of methanol solution was added to mix, and reacted at room temperature for 2 h. The sample was analyzed by HPLC (Luna^®^ LC C18 column 250 × 4.6 mm 5 μm, 1220 Infinity LC, Agilent Technologies, Santa Clara, CA, USA), detected with UV−Vis detector at 237 nm, and the following solvent system was used: methanol/0.3% phosphoric acid solution (75/25), isocratic elution at a flow rate of 1 mL/min; injection volume was 10 μL; analysis for 30 min. Processed cultivation product was analyzed by tandem LC-MS/MS, using an HPLC system comprising an Agilent 1200 HPLC system equipped with column (phenomenex C18 250 × 4.6 mm 5 μm), Diode Array Detector (DAD, Agilent), and ultra-high-resolution TOF mass detector (Bruker maXis, Bruker, Karlsruhe, Germany). The mobile phases were water plus 0.1% formic acid (solution A) and methanol solution (solution B) at 1 mL/min, at the wavelength of 237 nm to detect the compounds and recorded the mass spectra in the positive ion mode within 30 min.

### 2.6. Validation of MJ Pathway Gene Expression via Reverse Transcription PCR (RT-PCR)

*A. niger* transformants were cultivated for 3 days, the thallus collected by filtration, washed twice with sterile water, washed twice with 0.8 M NaOH, and ground with liquid nitrogen. Total RNA was isolated using RNAisoTM Plus (TaKaRa, Kusatsu, Japan) according to the manufacturer’s instructions. Reverse transcription was performed using the Evo M-MLV RT-PCR Kit (Accurate biotechnology, Changsha, China). Related primers are shown in Supplemental Table S2. Transcriptional expression levels of related genes were compared with *gpdA* (An16g01830). PCR amplification was programmed as follows: 94 °C for 30 s, 60 °C for 30 s, and 72 °C for 20 s, 25 cycles.

## 3. Results

### 3.1. Expression Cassette Assembly of MJ Biosynthetic Genes via 2μ Homologous Recombination

**Figure 2:**
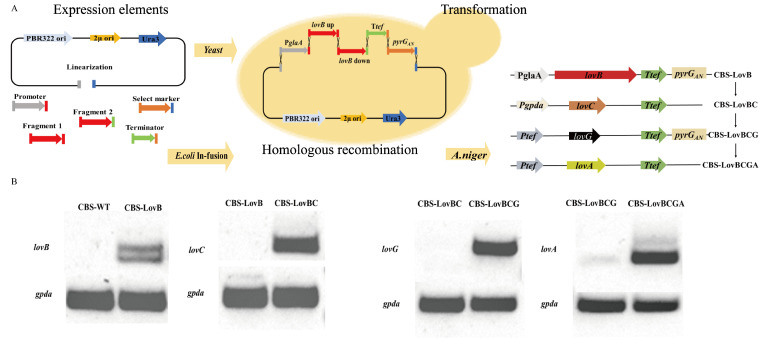
Construction of MJ biosynthetic pathway in *A. niger*. (**A**) Construction of MJ biosynthetic gene expression cassette via yeast 2μ homologous recombination and transformation of *A. niger* strains. (**B**) RT-PCR analysis of the expression of MJ genes in recombinant *A. niger* strains. *gpda* was used as the internal reference gene.

Firstly, we successfully constructed the integration expression cassettes for *lovC* (P*gpda*-*lovC*-T*tef*), *lovG* (P*tef*-*lovG*-T*tef*), and *lovA* (P*tef*-*lovA*-T*tef*) via the in-fusion method [23] in *E. coli*. As for *lovB*, a type I PKS gene containing multiple functional catalytic domains (KS, AT, DH, ER, KR, and ACP) [24], is too long (encode 335 kDa nonaketide synthase) to construct its expression cassette by the conventional method. To solve this problem, we used *Saccharomyces cerevisiae* 2μ homologous recombination to construct the expression cassette of *lovB* (P*glaA*-*lovB*-T*tef*) (Figure 2A). Only a 30–50-bp-long homologous region is needed between two DNA fragments, and these fragments can be connected via homologous recombination. Gibson et al. reported that 25 large overlapping DNA fragments were assembled to form complete *Mycoplasma* genome via one-step homologous recombination in yeast cells [25]. We assembled a linearized yeast expression vector and two fragments of *lovB* genes to construct *lovB* expression cassette via 2μ homologous recombination. Through yeast colony PCR identification and sequencing results, LovB expression cassette pYEP352-P*glaA*-*lovB*-T*tef*-*pyrG_An_* was successfully constructed.

### 3.2. Reconstruction of MJ Biosynthetic Pathway in A. niger

*A. niger* strain CBS-LovB was constructed by homologous recombination technology in glucoamylase site (An03g06550), with the integration of *lovB* expression cassette and *pyrG_An_* expression cassette. Through CRISPR/Cas9-HDR technology, the *lovC* expression cassette was integrated in *pyrG_An_* site of strain CBS-LovB, obtaining strain CBS-LovBC. In the same way, *lovG* and *lovA* was integrated in the gene site of *aamA* and *amyA* site. Finally, we constructed *A. niger* strain CBS-LovBCGA, which contains all of the MJ biosynthetic genes.

To determine the expression of the MJ biosynthetic genes, we used cDNA as the template of RT-PCR, and amplificated for 25 cycles. The results showed that all of the MJ biosynthetic genes (*lovB*/*C*/*G*/*A*) were successfully transcribed in *A. niger* strains compared to the control strain (Figure 2B and Supplemental Figure S2).

### 3.3. Synthesis of MJ in A. niger CBS-LovBCGA

**Figure 3:**
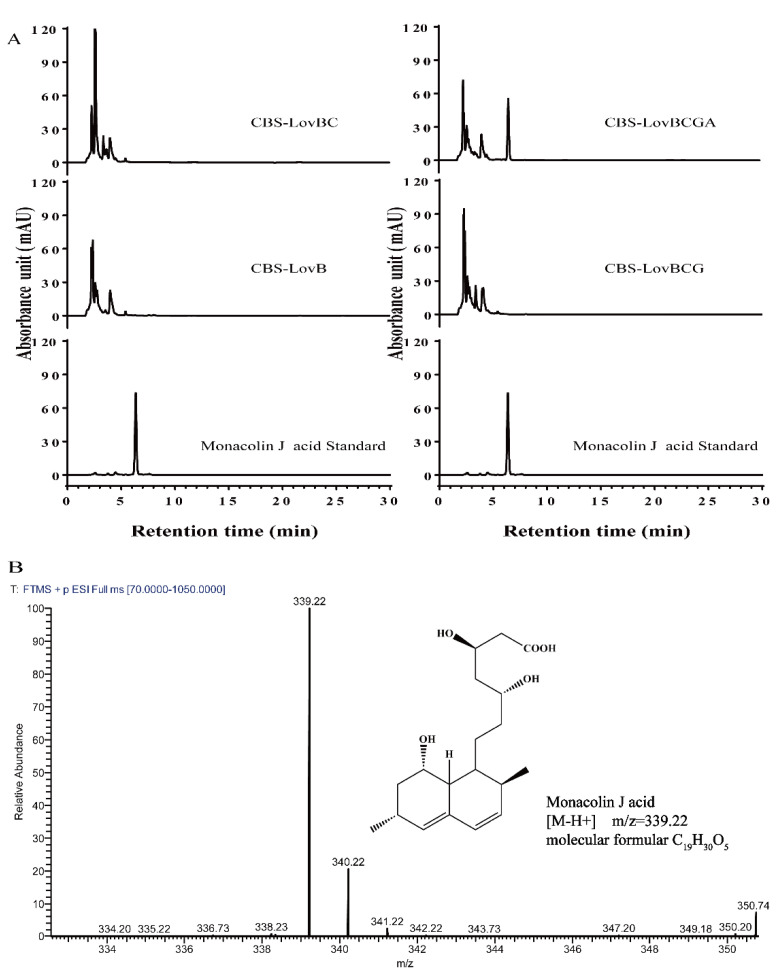
HPLC and LC-HR-ESIMS analysis of the heterologous production of MJ in *A. niger*. (**A**) HPLC detection of MJ in the cultivation sample of engineered *A. niger* strains. (**B**) LC-HR-ESIMS analysis of MJ in *A. niger* strain CBS-LovBCGA.

To detect the synthesis of MJ and its intermediate compound, *A. niger* strains (CBS-LovB, CBS-LovBC, CBS-LovBCG, and CBS-LovBCGA) were cultivated in DPY medium. After 7-day cultivation, the cultivation broth was extracted and analyzed. HPLC results showed that *A.niger* wildtype strain did not produce MJ and Lovastatin (Figure S3). When *lovB*, *lovC* and *lovG* genes were integrated into the genome of *A. niger* the strain CBS-LovBCG could produce dihydromonacolin L(DML). According to its chemical structure, DML, as a pyranone with no conjugated double bonds, did not show obvious ultraviolet absorption; LC-MS with an ELSD detector was used to detect DML. The results showed that DML was successfully produced in the strain CBS-LovBCG with a retention time of 4.81 min (Figure S4A) and a mass of 325.24 (Figure S4B). It has been reported that in the Lovastatin biosynthesis pathway, cytochrome P450 (LovA) catalyzed the double oxidation of the reaction from DML acid to MJ acid [26]. The HPLC results showed that strain CBS-LovBCGA could produce MJ with a retention time of 6.486 min (Figure 3A), which is consistent with the MJ standard sample (retention time of 6.473 min), while there was no corresponding product in the strains CBS-LovB, CBS-LovBC or CBS-LovBCG. The cultivation sample was further analyzed by LC-HR-ESIMS, and MJ acid was detected with the formula of C_19_H_30_O_5_ and an exact mass of 339.22 ([M-H]^+^) (Figure 3B). In addition, tandem LC-MS/MS analysis showed that the fragmentation spectra of the candidate compound produced by CBS-LovBCGA were identical to those of the MJ standard (Supplemental Figure S5). Taken together, a heterologous MJ biosynthetic pathway was successfully constructed in *A. niger* and we obtained an engineered strain that could directly produce MJ.

### 3.4. Optimization of Cultivation Conditions to Increase the Production of MJ

**Figure 4:**
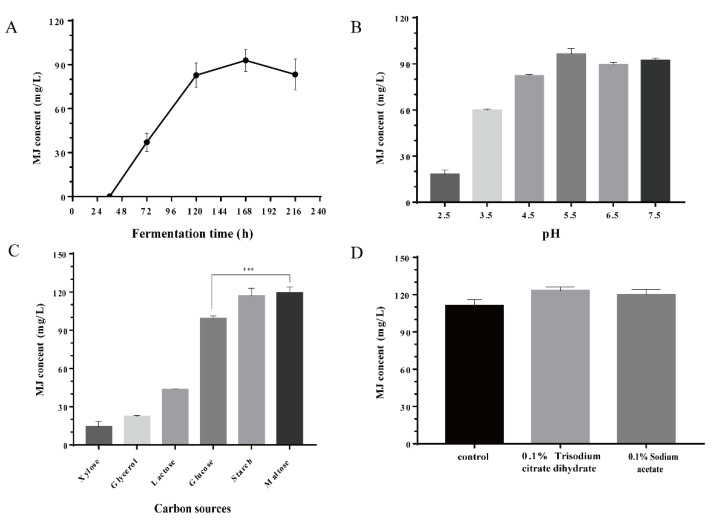
Optimization of shake flask cultivation for MJ production. The MJ titer was measured by HPLC. (**A**) The cultivation process curve of MJ production when glucose was used as carbon source. The initial pH of medium was 6.5. (**B**) MJ production after 7-day cultivation under different initial pH conditions. (**C**) MJ production after 7-day cultivation using different carbon sources. ***: *p*-value < 0.001. (**D**) Effects of exogenous addition of precursors on MJ production. Control: CBS-LovBCGA, no precursor addition.

For the quantification of MJ production in the engineered *A. niger* strain, the calibration curve of MJ was developed by HPLC (Figure S6). For MJ production, glucose was firstly selected as the carbon source. After a 7-day shake-flask culture, the highest titer of monacolin J reached 92.90 mg/L (Figure 4A). To determine the optimal pH for MJ production, cultivation was conducted under different initial pH ranging from 2.5 to 7.5. The results showed that pH 5.5 is the optimal pH for MJ cultivation, with the yield of MJ reaching 96.34 mg/L (Figure 4B), and the level of MJ was stable in the pH range of 5.5–7.5. According to a previous study, the acidic environment adversely affected the growth of the strain and the synthesis of enzymes in the lovastatin pathway [27]. Furthermore, during the metabolic process, various intermediate metabolites could be produced, resulting in a change in pH. Therefore, it was necessary to detect the pH during the whole process of MJ production.

It has been reported that delayed carbon sources such as maltodextrin and lactose are better able to increase the production of lovastatin [28]. To further improve the production of MJ, delayed carbon sources such as lactose, xylose, glycerol, starch and maltose were used for MJ production. The results showed that starch and maltose were more efficient carbon sources for MJ production than glucose. The highest yield reached 119.31 mg/L when maltose was used as the carbon source, which was an increase of 20.24% compared with MJ production with glucose (Figure 4C), while xylose sharply repressed the production of MJ. For lovastatin production, short-chain fatty acids such as acetic acid, as inducers of acetyl-CoA and propionyl-CoA synthases, could promote the synthesis of lovastatin in *A. terreus*, and the exogenous addition of sodium acetate and trisodium citrate dihydrate showed obvious promoting effect [29]. In this study, by adding different concentrations of sodium acetate and trisodium citrate dihydrate, the production of MJ was improved. When the concentration of trisodium citrate dihydrate was 0.1%, the titer of MJ reached 127.07 mg/L, increasing by 14.4%. Additionally, by adding 0.1% sodium acetate exogenously, the titer of MJ was 120.00 mg/L, an increase of 8.02% (Figure 4D).

### 3.5. Fed-Batch Cultivation by Adding Maltose for MJ Production

**Figure 5:**
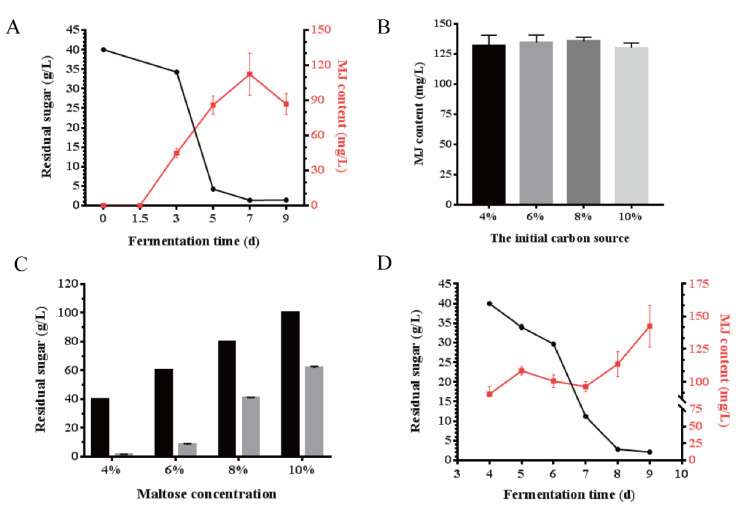
Fed-batch cultivation by adding maltose for MJ production. (**A**) Hyperbola of residual sugar content (black line, dot marker) and MJ yield during cultivation (red line, square marker). (**B**) The titer of MJ under different initial sugar content after 9-day cultivation. (**C**) Residual sugar content in medium with different initial sugar content. Black represents the initial sugar content, grey represents the residual sugar content. (**D**) Hyperbola of residual sugar content (black line, dot marker) and MJ titer in fed-batch cultivation (red line, square marker). Sugar content was added to the initial sugar content from the 4th day of cultivation.

To investigate the relationship between residual sugar content and MJ yield, we tested the residual sugar content in the course of MJ production by the phenol-sulfuric acid method [30] (the calibration curve is shown in Figure S7). The results showed that the carbon source in the culture medium was almost exhausted at the end. We speculated that due to insufficient sugar content in the medium at the late stage of cultivation, MJ production dropped (Figure 5A).

To resolve the insufficient carbon source at the late stage of MJ production, we firstly increased the initial sugar level from 4% to 6%, 8%, and 10%. However, after 9-day cultivation, there was no increase in MJ yield (Figure 5B). We found that residual sugar content increased in the late stage of MJ production when a high initial carbon source was provided in the medium (Figure 5C). In the early cultivation stage, high sugar content could promote mycelium growth, and raw materials such as acetyl-CoA were consumed vigorously, resulting in insufficient accumulation of secondary metabolites. While in the late cultivation stage, high sugar content perhaps inhibited the synthesis of MJ due to the glucose repression effect.

To further improve the production of MJ, sugar supplementation was carried out when sugar content decreased to half of the initial content (about 4 days, Figure 5A), and sugar content was added to the initial sugar content (40 g/L). The results showed that residual sugar content decreased more slowly than the cultivation process without sugar addition (Figure 5D). This kind of cultivation state was advantageous for maintaining mycelium growth and accumulate metabolites. The final MJ yield was increased to 142.61 mg/L (improved by 21.2%, Figure 5D) compared to MJ production without sugar supplementation.

## 4. Discussion

In this study, *Aspergillus niger* was selected as the host for the heterologous reconstruction of the MJ biosynthetic pathway due to its several advantages. Firstly, as a GRAS (Generally Recognized as Safe) strain, the *A. niger* host is safer and more reliable for manufacturing medicines in industrial production. Secondly, compared with the heterologous synthesis of MJ in yeast, heterologous synthesis of MJ in *A. niger* does not need to express phosphopantetheinyl transferase (PPTase) [31], which must be integrated into the yeast to catalyze the attachment of a 4′-phosphopantetheine moiety derived from coenzyme A to PKS and NRPS and transform them from an inactive apo-form to an active holo-form [32]. Additionally, it could accurately heterologously express the fungal BGCs (biosynthetic gene clusters) without removing introns, and minimize the impact of incorrect intron splicing in heterologous expression [33]. Thirdly, compared with other heterologous hosts, *A. niger* has certain advantages in the production of secreted MJ. It has been reported that the production of lovastatin in wild strains of *A. terreus* ATCC20542 shake flask fermentation was 400 μg/mL [34], with the industrial production strains mainly being obtained by screening through mutagenesis breeding. As for heterologous expression, Tang et al. successfully assembled the biosynthetic pathway with LovB, LovC, LovG, LovA, and cytochrome P450 reductase gene (CPR) from *A. terreus* in *S. cerevisiae* BJ5464-NpgA, and the production of 20 mg/L lovastatin [12] was further optimized by increasing the copy number of the *lovA* gene and developing an in situ cell lysis process resulting in theproduction of 55 mg/L simvastatin. In another report, Liu et al. reconstructed the biosynthetic pathway in *P. pastoris*, producing 60 mg/L MJ and 14.4 mg/L lovastatin. After a series of optimizations of fermentation conditions and co-culture strategies, MJ production of 593.9 mg/L was achieved on methanol [11].

In the existing fungal heterologous expression systems, constitutive promoters derived from housekeeping genes or inducible promoters from primary metabolic genes are frequently used to drive the expression of BGC genes [35]. In the present study, inducible promoters of glucoamylase (*glaA*), constitutive strong promoters of transcription elongation factor (*tef1*), and 3-phosphate glycerol dehydrogenase (*gpd*) were used in the expression cassettes of construction. As for the MJ biosynthetic strain CBS-LovBCGA, endogenous sugar-utilizing genes including *glaA*, *aamA* and *amyA* were knocked out, and therefore delayed carbon sources (such as starch and maltose) could be used for MJ production, which could activate and improve the production of MJ [36]. To further improve the production of MJ, CRISPR/Cas9 technology was applied to the multi-integrate *lovA* gene, which may enhance the oxidation process of DML to MJ. We obtained a double-copy *lovA* strain to compare with CBS-LovBCGA. However, the results showed that a higher expression level of the double-copy *lovA* did not significantly increase the production of MJ (data not shown).

Taken together, we constructed an effective MJ heterologous biosynthetic system in *A. niger*, which could provide a possible strategy for the production of other valuable compounds. In future work, to further improve the production level of MJ, precursor (such as acetyl-CoA and malonyl-CoA) supply could be an important strategy for improving the production of MJ derivative statins such as lovastatin [37,38] and simvastatin [39].

## 5. Conclusions

In this study, the MJ biosynthetic pathway genes (*lovB*, *lovC*, *lovG* and *lovA*) were heterologously integrated into the genome of *A. niger* CBS513.88 with strong promoters and suitable integration sites via yeast 2μ homologous recombination to create recombinant DNA and CRISPR-HDR technology for site-directed integration. The production of MJ (339.22 [M-H]^+^) was achieved, with the yield of MJ reaching 92.90 mg/L after 7-day cultivation. By optimizing the cultivation conditions and adding precursor, the final titer of MJ reached 142.61 mg/L on the fourth day of fed-batch cultivation, which represents an increase of 53.5% compared to the initial growth conditions. The heterologous biosynthesis of MJ could avoid the cumbersome process of producing MJ by alkali hydrolysis of lovastatin and industrial pollution. The obtained engineered *A. niger* MJ-producing strain could be further used to construct the heterologous biosynthetic pathway for derivative statin compounds.

## Figures and Tables

**Table 1:** Strains used in this study.

A. *niger* Strain	Parental Strain	Description	Expression Cassette	Reference
CBS-WT	A. *niger* CBS513.88	Δ*kusA*; Δ*pyrG*	-	Constructed in our lab
CBS-LovB	CBS-WT	P*glaA*-*lovB*-T*tef*-*pyrG_An_*	*lovB*	this study
CBS-LovBC	CBS-LovB	P*gpda*-*lovC*-T*tef*	*lovB*, *lovC*	this study
CBS-LovBCG	CBS-LovBC	P*tef*-*lovG*-T*tef*-*pyrG_An_*	*lovB*, *lovC*, *lovG*	this study
CBS-LovBCGA	CBS-LovBCG	P*tef*-*lovA*-T*tef*	*lovB*, *lovC*, *lovG*, *lovA*	this study

## Supplementary Materials

The following supporting information can be downloaded at: https://www.mdpi.com/article/10.3390/jof8040407/s1, Figure S1: Schematic of *Aspergillus niger* integration site construction; Figure S2. RT-PCR analysis of the expression of MJ genes in *A. niger* CBS-WT and CBS-LovBCGA strain; Figure S3: HPLC identification of *A. niger* strain fermentation product and standard products at 237 nm wavelength; Figure S4: LC-MS identification DML acid of *A. niger* transformated strain CBS-LovBCG fermentation product; Figure S5. Tandem LC-MS/MS analysis of the mass spectra fragmentation of MJ standard (A) and MJ produced by *A. niger* strain CBS-LovBCGA (B); Figure S6: Calibration curves of Monacolin J developed by HPLC; Figure S7: Calibration curves of the concent of Maltose; Table S1. The plasmid construction strategy used in this study; Table S2: Construction and identification of primers used in this study; Table S3. CRISPR/Cas9 plasmids used in this study.
