# Supplementary material for: Heterologous Synthesis of Monacolin J by Reconstructing Its Biosynthetic Gene Cluster in Aspergillus niger
Source: J Fungi (Basel). 2022 Apr 16;8(4):407. doi: 10.3390/jof8040407 (PMC9032219; doi:10.3390/jof8040407)
Supplement: Supplementary file 1 [file jof-08-00407-s001.zip › jof-1645485-supplementary.pdf]

Supporting Information

Heterologous synthesis of Monacolin J by reconstructing its biosynthetic gene cluster in *Aspergillus niger*

Xu Zeng [1], Junwei Zheng [1], Feifei Lu[1], Li Pan[1 2*], Bin Wang [1 2*]

1 School of Biology and Biological Engineering, South China University of Technology, Guangzhou Higher Education Mega Center, Panyu town Guangzhou 510006, China,

2 Guangdong Provincial Key Laboratory of Fermentation and Enzyme Engineering, Guangzhou 510006, China

* Correspondence: btbinwang@scut.edu.cn (B.W) btlipan@scut.edu.cn (L.P)

Supplemental Table S1. The plasmid construction strategy used in this study

Fragments assembled by overlapping PCR (Corresponding primers)			Generated plasmid via in-fusion cloning
**Fragment A**	**Fragment B**	**Fragment C**	
*Up_{pyrG}-P_{gpdA} (Up_{pyrG}-F/R, P_{gpdA}-F/R)	*LovC* (*lovC*-F/R)	T_{tef}-*Dn_{pyrG} (T_{tef}-1-F/R, Dn_{pyrG}-F/R)	pM18T-Up_{pyrG}-P_{gpdA}-*lovC*-T_{tef}-Dn_{pyrG}
Up_{aamA}-P_{tef} (Up_{aamA}-F/R, P_{tef}-1-F/R)	*LovG* (*lovG*-F/R)	T_{tef}-*pyrG*$_{An}$-Dn_{aamA} (T_{tef}-2-F/R, *pyrG*$_{An}$-F/R, Dn_{aamA}-F/R)	pM18T-Up_{aamA}-P_{tef}-*lovG*-T_{tef}-*pyrG*$_{An}$-Dn_{aamA}
Up_{amyA}-P_{tef} (Up_{amyA}-F/R, P_{tef}-2-F/R)	*lovA* (*lovA*-F/R)	T_{tef}-Dn_{amyA} (T_{tef}-3-F/R, Dn_{amyA}-F/R)	pM18T-Up_{amyA}-P_{tef}-*lovA*-T_{tef}-Dn_{amyA}

*Up_{geneA}: represents the upstream region of gene A.
Dn_{geneA}: represents the downstream region of gene A.

Supplemental Table S2. Construction and identification primers used in this study

Primer	Sequences	Function description
lovB-up-F	ACTGAGAGCCTGAGCTTCATCCCCAGCATC ATTACACCTCAGCATCTAGAATGGCTCAAT CTATGTATCCTAATG	Amplification of the upstream fragment of *lovB* ORF
lovB-up-R	GCCGGTCCCTGCTCCAATCTCCAGAATAT CCATTGACTGATAGCGATGGGCGATCTGC GCCACCAATTCCCGGGCGTAGT	Amplification of the upstream fragment of *lovB* ORF
lovB-dn-F	ACTACGCCCGGGAATTGGTGGCGCAGATC GCCCATCGCTATCAGTCAATGGATATTCTG GAGATTGGAGCAG	Amplification of the downstream fragment of *lovB* ORF
lovB-dn-R	TCGTAAAGGCTTTTTAAGGAAGTCATAA CGGCATAAATCGAATGTCCGCTCATGCCA GCTTCAGGGCGG	Amplification of the downstream fragment of *lovB* ORF
Up_{pyrG}-F	ACCCGGGGATCCTCTAGAGACTGGTGGC AGGCGTCAAGT	Amplification of the upstream fragment of $pyrG_{An}$
Up_{pyrG}-R	CTCTTGGGTCTCTCCCGTCAGATGTTAAA GGGTTGGGATGG	Amplification of the upstream fragment of $pyrG_{An}$
P_{gpdA}-F	CATCCCAACCCTTTAACATCTGACGGGAG AGACCCAAGAG	Amplification of the fragment P_{gpdA}
P_{gpdA}-R	ATGAATGGCTGGTCGCCCATTGTTTAGATG TGTCTATGTGGCG	Amplification of the fragment P_{gpdA}
lovC-F	CACATAGACACATCTAAACAATGGG CGACCAGCCATTCATT	Amplification of the whole *lovC* ORF
lovC-R	TAGCGAAATGGATTGATTGTTTACGG CCCCTCGAGCCGA	Amplification of the whole *lovC* ORF
T_{tef}-1-F	TTCGGCTCGAGGGGCCGTAAGCGGACATT CGATTTATGCCGTT	Amplification of the fragment T_{tef}
T_{tef}-1-R	GACGTTGGTTTTCTTCTCCTGTATTGGGAT GAATTTTGTATGCAC	Amplification of the fragment T_{tef}
Dn_{pyrG}-F	TACAAAATTCATCCCAATACAGGAGAAG AAAACCAACGTCAC	Amplification of the downstream fragment of $pyrG_{An}$
Dn_{pyrG}-R	CTTGCATGCCTGCAGGTCGACGATAACTC AACCGAGCGTGCCGT	Amplification of the downstream fragment of $pyrG_{An}$
Up_{aamA}-F	ACCCGGGGATCCTCTAGAGAGCGTT GTTTCTTACCCCC	Amplification of the upstream fragment of Up_{aamA}
Up_{aamA}-R	GCGGTGATTCTGCTGTCTCGGAATTG TCCGTCCTACCGAA	Amplification of the upstream fragment of Up_{aamA}

P_{tef}-1-F	TTCGGTAGGACGGACAATTCCGAGACAGCAGAATCACCGC	Amplification of the fragment P_{tef}
P_{tef}-1-R	GGAGATGCTTGGTAACGCATGTGAAGGTTGTGTTATGTTTTGTG	Amplification of the fragment P_{tef}
lovG-F	AAACATAACACAACCTTCACATGCGTTACCAAGCATCTCCA	Amplification of the whole *lovG* ORF
lovG-R	GGCATAAATCGAATGTCCGCCTACTCCAATGTCTGGGCCG	Amplification of the whole *lovG* ORF
T_{tef}-2-F	CGGCCCAGACATTGGAGTAGGCGGACATTCGATTTATGCCGTT	Amplification of the fragment T_{tef}
T_{tef}-2-R	CGCGTTCTCGAGGAAGTTGCGTATTGGGATGAATTTTGTATGCA	Amplification of the fragment T_{tef}
$pyrG_{An}$-F	TACAAAATTCATCCCAATACGCAACTTCCTCGAGAACGCG	Amplification of the fragment $pyrG_{An}$
$pyrG_{An}$-R	CCAACGTACTTGGTCACCCGCCCTTTTAGTCAATACCGTTAC	Amplification of the fragment $pyrG_{An}$
Dn_{aamA}-F	AACGGTATTGACTAAAAGGGCGGGTGACCAAGTACGTTG	Amplification of the downstream fragment of *aamA*
Dn_{aamA}-R	ATGCCTGCAGGTCGACGATAGCCCTGGTACAGTTAGCG	Amplification of the downstream fragment of *aamA*
Up_{amyA}-F	ACCCGGGGATCCTCTAGAGAGCAATCCATTTATTTCCTTCTC	Amplification of the upstream fragment of Up_{amyA}
Up_{amyA}-R	GCGGTGATTCTGCTGTCTCGGAGCGCCTTCAAGTCATCT	Amplification of the upstream fragment of Up_{amyA}
P_{tef}-2-F	CAGATGACTTGAAGGCGCTCCGAGACAGCAGAATCACCGC	Amplification of the fragment P_{tef}
P_{tef}-2-R	TGAGCGCGTCGACAGTCATGGTGAAGGTTGTGTTATGTTTTGTG	Amplification of the fragment P_{tef}
lovA-F	AACATAACACAACCTTCACCATGACTGTCGACGCGCTCACA	Amplification of the whole *lovA* ORF
lovA-R	GGCATAAATCGAATGTCCGCCTATAGTGAACCAGGAAGGCGG	Amplification of the whole *lovA* ORF
T_{tef}-3-F	GCCTTCCTGGTTCACTATAGGCGGACATTCGATTTATGCCGTT	Amplification of the fragment T_{tef}
T_{tef}-3-R	CGACCATAAGATACATCCCCGTATTGGGATGAATTTTGTATGCA	Amplification of the fragment T_{tef}
Dn_{amyA}-F	TACAAAATTCATCCCAATACGGGGATGTATCTTATGGTCG	Amplification of the downstream fragment of *amyA*
Dn_{amyA}-R	ATGCCTGCAGGTCGACGATACGGAT	Amplification of the

	CACCGTCGAGCAC	downstream fragment of *amyA*
LovB-YZ1-F	CGTACCTGGCGACCTATGACTA	Yeast transformation verification primers
LovB-YZ1-R	GTAGGTAGCCTTGATGAGTGCC	Yeast transformation verification primers
LovB-YZ2-F	AGGGAGACTACTTGAGCGGTGA	Yeast transformation verification primers
LovB-YZ2-R	TGAACACTCTCAGGCACAAACC	Yeast transformation verification primers
LovB-YZ3-F	ACTGTCTCTCGGGCAAGAATAC	Yeast transformation verification primers
LovB-YZ3-R	TCAACTCTCCCCTCTGTGCTAA	Yeast transformation verification primers
LovB-JD-1-F	GAGGATTGCCTGAACATTGACA	Location of *lovB* in the *glaA* (An03g06550) locus
LovB-JD-1-R	ACTCACGCCTCTGGCATAGC	Location of *lovB* in the *glaA* (An03g06550) locus
LovB-JD-2-F	TAACATCTAAACTCTTCTCCATCG	Location of *lovB* in the *glaA* (An03g06550) locus
LovB-JD-2-R	ACGGTCTGGTTCTGTAGTTCC	Location of *lovB* in the *glaA* (An03g06550) locus
LovC-JD-1-F	CGGAAAGTGGAACGATTGGGGG	Location of *lovC* in the $pyrG_{An}$ locus
LovC-JD-1-R	TGGCAAAGGCAGCCCAAGCAAC	Location of *lovC* in the $pyrG_{An}$ locus
LovC-JD-2-F	GCAGTGAGGAAGAGCGGCAG	Location of *lovC* in the $pyrG_{An}$ locus
LovC-JD-2-R	ATTGTCGATCTGCCCACTTTCT	Location of *lovC* in the $pyrG_{An}$ locus
LovG-JD-1-F	CGTCCTTCGGTCGGAATGATG	Location of *lovG* in the *aamA* (An11g03340) locus
LovG-JD-1-R	CGCTCACGGAGGGTCCATTAT	Location of *lovG* in the *aamA* (An11g03340) locus
LovG-JD-2-F	GGAACGAAGTGATGAAGAACCA	Location of *lovG* in the *aamA* (An11g03340) locus
LovG-JD-2-R	CATACGAGGCGAAACGGGGA	Location of *LovG* in the *aamA* (An11g03340) locus
LovA-JD-1-F	ATATCAGGCCGCGCACGAAAG	Location of *lovA* in the *amyA* (An05g02100) locus
LovA-JD-1-R	CCTCATGGTCGTCAGTTCCC	Location of *lovA* in the *amyA* (An05g02100) locus
LovA-JD-2-F	TTCGGTTGGCATCCACGGGC	Location of *lovA* in the *amyA* (An05g02100) locus

LovA-JD-2-R	TTAACAGTTTGCGGGAGATGCA	Location of *lovA* in the *amyA* (An05g02100) locus
RT-*lovB*–F	GCGTCCAACCGCATCTCG	Semi-quantitative primers for *lovB* gene
RT-*lovB*–R	AGATTCGCACCCGCAGCAA	Semi-quantitative primers for *lovB* gene
RT-*lovC*–F	ACCGCTGGATTGGCGATGA	Semi-quantitative primers for *lovC* gene
RT-*LovC*–R	CGATAGTCAAAGACCTCCTC	Semi-quantitative primers for *lovC* gene
RT-*lovG*–F	CGACAACAATCATAATGGACCC	Semi-quantitative primers for *lovG* gene
RT-*lovG*–R	CTTGCCTCGTCTTGATACCAG	Semi-quantitative primers for *lovG* gene
RT-*lovA*–F	CTGGTTCTCGCAAAATGATAAG	Semi-quantitative primers for *lovA* gene
RT-*lovA*–R	GTGACTTCCTTGAATCCATCG	Semi-quantitative primers for *lovA* gene
RT-*gpdA*-F	CTCTGCTCCTTCCGCTGAT	Semi-quantitative primers of internal reference gene *gpda* (An16g01830)
RT-*gpdA*-R	ACCCTCAACGATGCCGAAC	Semi-quantitative primers of internal reference gene *gpda* (An16g01830)

Supplemental Table S3. CRISPR/Cas9 plasmids used in this study

Plasmids	Description/derivation	Reference
PFC332-*pyrG$_{An}$*-*hygB*	PFC332 derivate with gRNA scaffold of *pyrG$_{An}$* under the control of promoter of *A. oryzae* U6 and *A. fumigatus* U6-2	This work
PFC332-*aamA*-*hygB*	PFC332 derivate with gRNA scaffold of *aamA* under the control of promoter of *A. oryzae* U6 and *A. fumigatus* U6-2	This work

PFC332-*aamA*-*amyA-hygB*	PFC332 derivate with gRNA scaffold of *aamA* and *amyA* under the control of promoter of *A. oryzae* U6 and *A. fumigatus* U6-2	This work

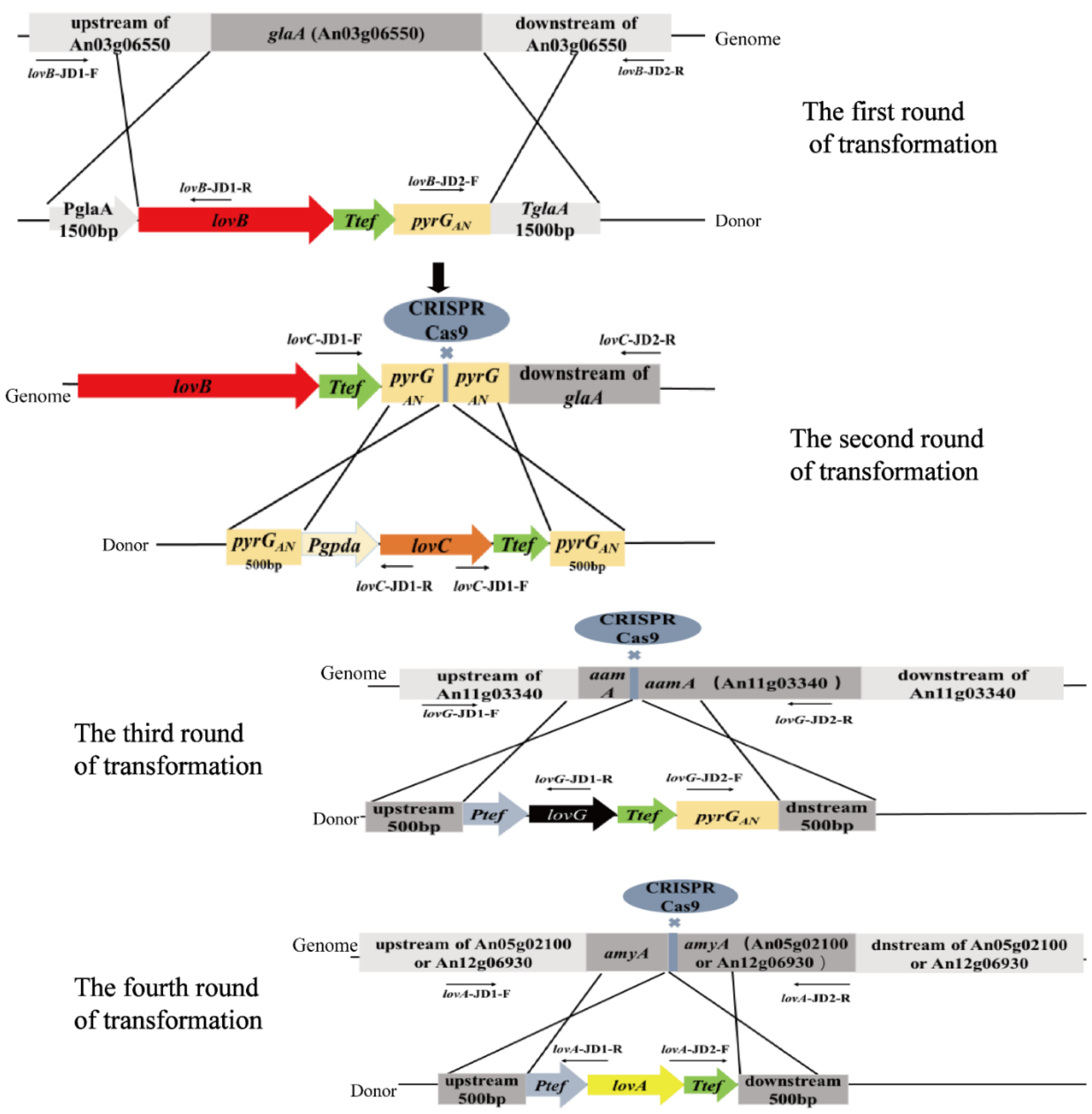

Figure S1. Schematic gene integration in *A. niger*.

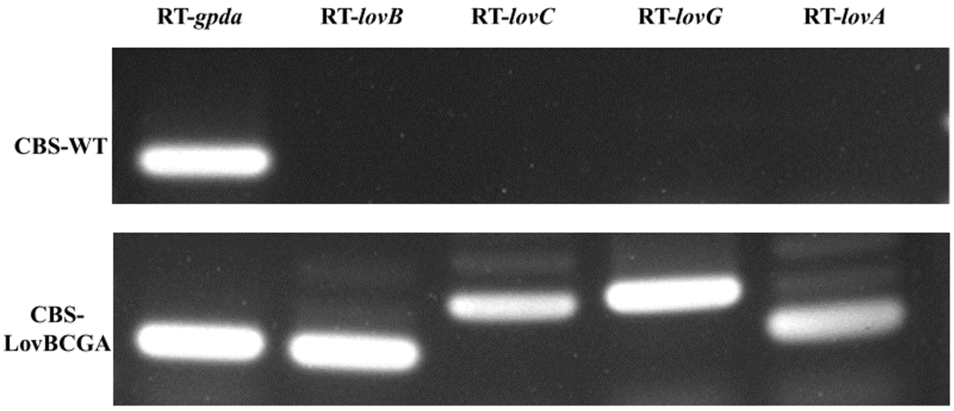

Figure S2. RT-PCR analysis of the expression of MJ genes in *A. niger* CBS-WT and CBS-LovBCGA strain. *gpda* was used as the internal reference gene.

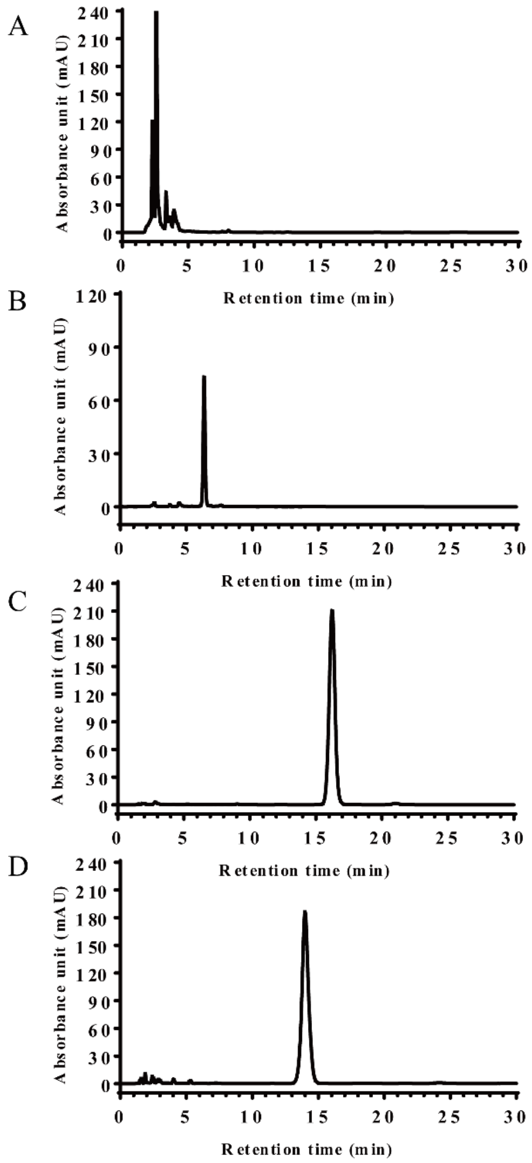

Figure S3. HPLC identification of *A. niger* strain fermentation product and standard products at 237 nm wavelength. (A) *A. niger* strain CBS513.88(Δ*kusA*, Δ*pyrG*) (B) Monacolin J acid standard, retention time 6.473 min (C) Lovastatin lactone standard, retention time 16.238 min (D) Lovastatin acid standard, retention time 13.999 min.

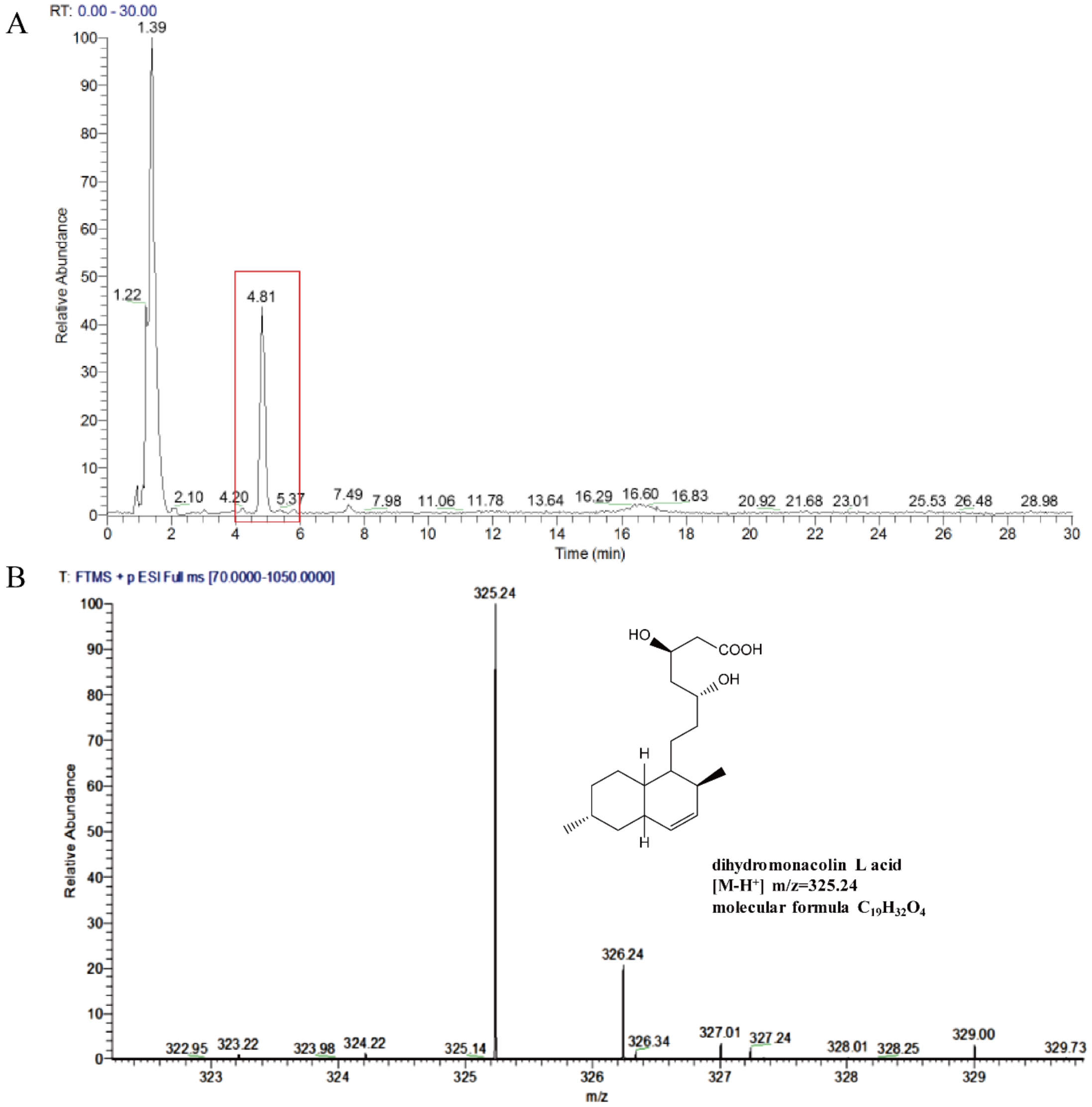

Figure S4. LC-MS identification DML acid of *A. niger* transformated strain CBS-LovBCG fermentation product (A) DML acid, retention time 4.81 min with the red square. (B) LC-MS identification of DML acid from *A. niger* strain CBS-LovBCG.

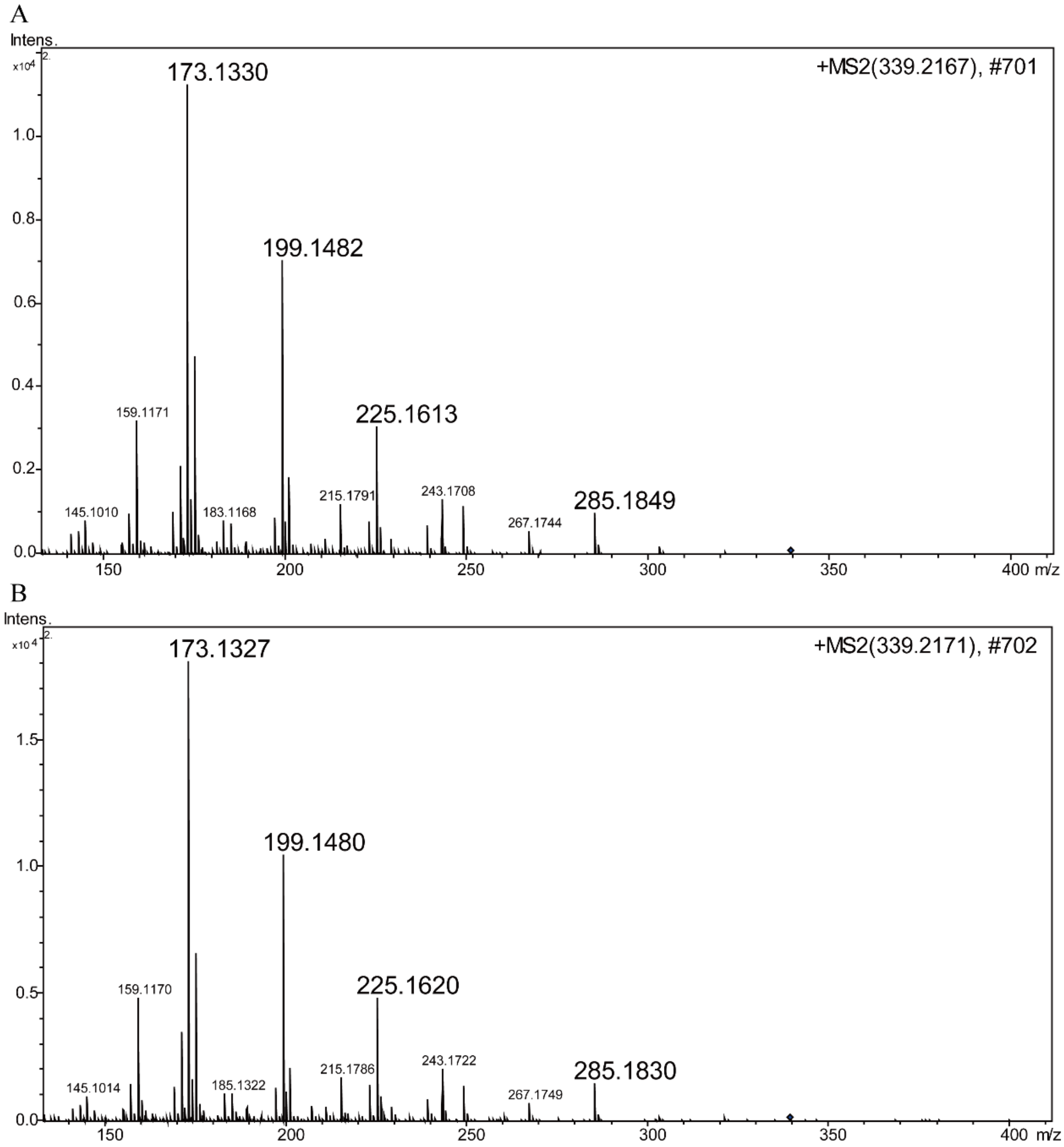

Figure S5. Tandem LC-MS/MS analysis of the mass spectra fragmentation of MJ standard (A) and MJ produced by *A. niger* strain CBS-LovBCGA (B).

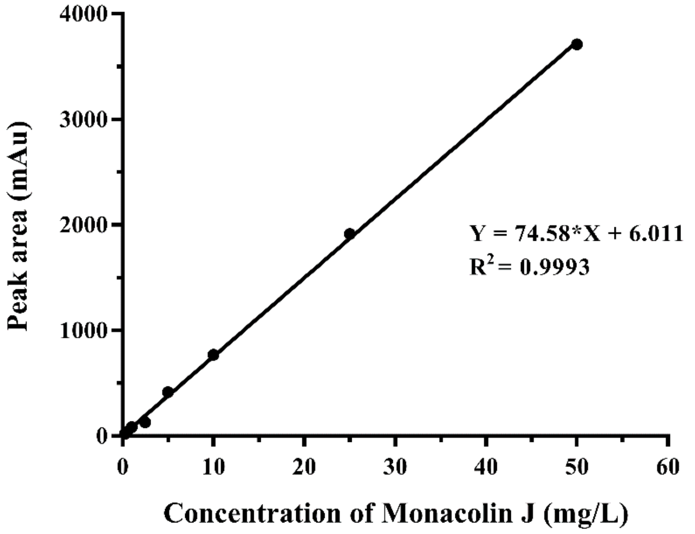

Figure S6. Calibration curves of Monacolin J developed by HPLC

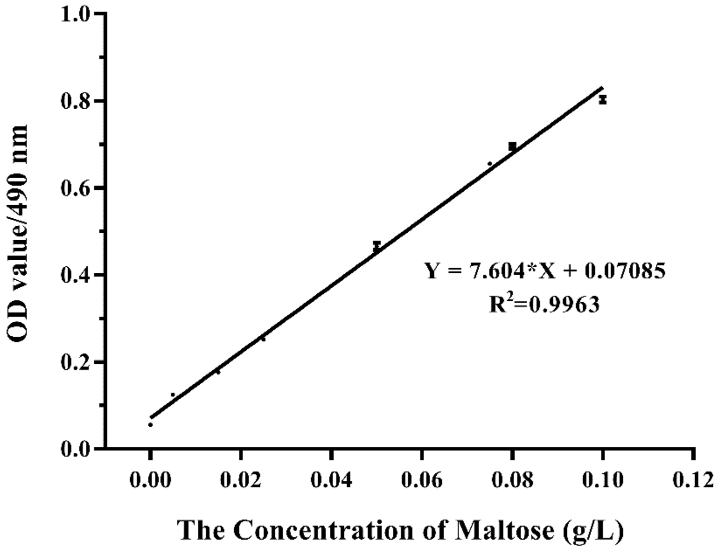

Figure S7. Calibration curves of the concentration of Maltose
